# *De Novo* Assembly of Transcriptome and Development of Novel EST-SSR Markers in *Rhododendron rex* Lévl. through Illumina Sequencing

**DOI:** 10.3389/fpls.2017.01664

**Published:** 2017-09-26

**Authors:** Yue Zhang, Xue Zhang, Yue-Hua Wang, Shi-Kang Shen

**Affiliations:** School of Life Sciences and Laboratory of Ecology and Evolutionary Biology, Yunnan University, Yunnan, China

**Keywords:** *Rhododendron rex*, EST-SSRs, transcriptome sequencing, genomics, evolutionary adaptation

## Abstract

Transcriptome sequences generated by next-generation sequencing (NGS) technologies can be utilized to rapidly detect and characterize a large number of gene-based microsatellites from different plants. *Rhododendron rex* Lévl. is a perennial woody species from the family Ericaceae and an endangered plant with high ornamental value endemic to Southwestern China. Nevertheless, the genetic and genomic information of *R. rex* remain unknown. In this study, we performed transcriptome sequencing for *R. rex* leaf samples, and generated large transcript sequences for functional characterization and development gene-associated SSR markers. A total of 164,242 unigenes were assembled and 115,089 (70.07%) unigenes were successfully annotated in public databases. In addition, a total of 15,314 potential EST-SSRs were identified, and the frequency of SSRs in the *R. rex* unigenes was 9.32%, with an average of one EST-SSR per 5.65 kb. The most abundant type was repeated di-nucleotide (54.63%), followed by mono- (26.03%) and tri-nucleotide (18.51%) repeats. Based on the SSR-containing sequence, 100 primer pairs were randomly selected and synthesized and used for assessment of the polymorphism. Thirty-six primer pairs were polymorphic and revealed polymorphism among 20 individuals from four *R. rex* populations. A total of 197 alleles were identified, with an average of 5.472 alleles per locus. The Polymorphism Information Content ranged from 0.154 to 0.870, with a mean of 0.482. The newly developed EST-SSR markers exhibited high transferability (58.33–83.33%) among the six subgenera. Thus, these novel EST-SSR markers developed would provide valuable sequence resources for population structure, genetic diversity analysis, and genetic resource assessments of *R. rex* and its related species.

## Introduction

*Rhododendron* L. is the largest woody plant genus, which contains at least 1,025 species and belongs to the Ericaceae family, and is widely distributed from the northern temperate zone, throughout tropical Southeast Asia, to northeastern Australia (Chamberlain et al., 1996). In China, more than 500 species of *Rhododendron* exist; of which, 70% are endemic and most species occur in the northwestern region of Yunnan provinces. Northwestern Yunnan has been recognized as a diversification and differentiation center of modern *Rhododendron* L. (Ma et al., 2013; Shen et al., 2015). *Rhododendron* species are not only the major components of alpine and subalpine vegetation but also recognized as famous woody ornamental plants worldwide (Yoichi et al., 2016). However, *Rhododendron* germplasm is at risk of extinction due to the high frequency of habitat disturbance and climate change (Wu et al., 2015). Thus, wild *Rhododendron* germplasm must be conserved to maintain the stability of ecosystems and promote sustainable resource utilization.

Next-generation sequencing (NGS), particularly *de novo* transcriptome sequencing, has become a cutting-edge method for high-throughput sequencing in life sciences research (Mardis, 2008; Davey et al., 2011; Zhou et al., 2016). NGS is an accurate and efficient technology with high throughput and low cost and thus can be used to discover comprehensive expressed sequence data for several non-model species. Transcriptome sequencing not only offers a simple and effective method of developing a large number of unigene-based SSR markers but also allows rapid and comprehensive analyses of the plant genome in many woody plants (Li et al., 2012; Tan et al., 2013; Chen L. Y. et al., 2015). NGS technologies have contributed to improvements in the fields of ecology, evolution, and conservation genetics by increasing the amount of large-scale genomic and transcriptomic data available for non-model woody plant species.

Many types of markers have been developed and used for genetic linkage, molecular-assisted breeding, genetic diversity analysis, and genetic quality detection (Paux et al., 2012; Wu et al., 2014). Simple sequence repeat (SSR) markers can be categorized into genomic SSRs, which are identified from random genomic sequences, and expressed sequence tag (EST) SSRs, which are identified from transcribed RNA sequences (Song et al., 2012). These tandem repeated sequences contain mono-, di-, tri-, tetra-, penta-, or hexa-nucleotide units, which possess abundant information, codominance, and loci specificity; as such, SSR markers are more convenient to use for detection compared with other molecular markers (Zhou et al., 2016). SSR makers have been widely used as a powerful tool in studies of population genetic diversity and genetic structure analysis (Yoichi et al., 2016). Inferring genetic variation patterns within a species is crucial to understand population structure, local adaptation, and diversity differences among populations (Marsden et al., 2012). Climatic/environmental factors and adaptive processes play an important role in the maintenance of lineage divergence of alpine and subalpine *Rhododendron* species (Cao et al., 2004; Hart, 2015). Thus, SSRs derived from expressed sequence tags (EST-SSRs) can be used in studies on local adaptation and effects of environmental variability because of the potential relationship of these tags to functional genes controlling a certain phenotype (Cordeiro et al., 2001; Varshney et al., 2005; Kumari et al., 2013). Moreover, EST-SSRs, which are developed from the transcribed regions in a genome, are more evolutionarily conserved and have higher levels of transferability to related species than non-coding genomic SSRs (Wei et al., 2011). However, few scholars have investigated the development of EST-SSRs through transcriptome sequencing analysis in *Rhododendron*, particularly for endemic species.

In this study, we used an Illumina sequencing platform for transcriptome sequencing analysis of RNA extracted from the leaves of *R. rex* Lévl., an endangered plant endemic to China (Fu and Jin, 1992). The sequencing data were assembled and annotated, and EST-SSR markers were also developed. Specifically, the aims of this study were to (1) characterize the transcriptome of *R. rex*; (2) determine the frequency and distribution of SSRs, develop EST-SSR markers and examine the level of polymorphism; (3) evaluate the transferability of the polymorphism EST-SSR markers to related species.

## Materials and methods

### Plant material and RNA isolation

Three *R. rex* plant materials were collected from the Baicaoling in Dayao County, Yunnan Province in October 2016 and sampled at least 15 m apart from each other. Fresh leaf samples were cleaned and immediately placed in liquid nitrogen until RNA extraction. For Illumina sequencing, total RNA was isolated from each sample by using TRIzol® Reagent (Invitrogen, CA, USA). RNA quality was verified using a Nanodrop 2000 (Thermo, USA). RNA Integrity Number value of the three samples were more than 7, as assessed using an Agilent 2100 Bioanalyzer (Agilent Technologies, CA, USA). The integrity of the qualified RNA was analyzed through agarose gel electrophoresis. More than 30 ng of RNA was equally pooled from the three individuals for preparing a complementary DNA (cDNA) library.

### cDNA library construction and sequencing

The cDNA library for mRNA-seq was prepared using Illumina Truseq™ RNA sample prep Kit (Illumina, CA, USA). The poly(A) tail structure of eukaryotic mRNA 3′ end was isolated via magnetic oligo (dT) beads. mRNA samples were randomly sheared for transcriptome information analysis, added with fragmentation buffer and broken into 300 bp fragments. The first-strand cDNA was formed via reverse transcription using reverse transcriptase and random hexamer primer using mRNA as a template. Then, second-strand cDNA was synthesized, forming a stable double-stranded structure. cDNA double-strand structure is cohesive end, and adding End Repair Mix results in a blunt ended structure. To select the proper templates for downstream enrichment, the products of the ligation reaction were purified on 2% agarose gel (Certified Low Range Ultra Agarose). PCR amplification of 15 cycles was conducted to enrich the purified cDNA template using PCR primers PE 1.0 and 2.0 (Illumina) using DNA polymerase. The cDNA library was sequenced using Illumina HiSeq4000 sequencing platform at the Major Company (Shanghai, China). The images were automatically collected by base calling generation into FASTQ files (.fq).

### Sequence assembly and annotation

By removing the adapter contaminants, reading too much poly-N, reading <20 bp to filter the raw sequencing data, screening out high-quality clean read data for *de novo* assembly, empty reads and reads with *Q* < 20, using the program SeqPrep (https://github.com/jstjohn/SeqPrep). The GC content, sequence replication levels, and Q20 and Q30 values of the obtained clean data were calculated (Grabherr et al., 2011). In addition, the resulting cleaning data is *de novo* assembled by the short reading assembly program Trinity (http://trinityrnaseq.sourceforge.net/). This soft combines reads that have overlapping nucleic acid sequences to form contigs. Then, obtained contigs are assembled and sequences that cannot be extended at both end are defined as unigenes. The assembled unigenes were aligned using BLASTX with the public database (*E* < 1e-5), such as NCBI Nr, String, Swissprot, Kyoto Encyclopedia of Genes, and Genomes (KEGG), and Pfam, to obtain protein function annotation and classification information (Camacho et al., 2009). The gene ontology (GO) annotation information of these unigenes were obtained from the NCBI Nr database by using the program Blast2GO and contains molecular functions, biological processes, and cellular components (Conesa et al., 2005). Furthermore, these unigenes were categorized and analyzed in the COG database (http://www.ncbi.nlm.nih.gov/COG/) based on the COG information obtained from the string. The program WEGO (Ye et al., 2006) classified all unigenes based on the GO annotation information.

### Detection of the new EST-SSR markers and primer design

Potential SSRs were detected using the program MISA version 1.0 (http://pgrc.ipk-gatersleben.de/misa/). The mono-, di-, tri-, tetra-, penta-, and hexa-nucleotides were designed with minimum repeat numbers of 10, 6, 5, 5, 5, and 5 for the SSRs, respectively. With default parameter values, EST-SSR primers were designed using the Primer 3 (http://probes.pw.usda.gov/cgi-bin/batchprimer3/batchprimer3.cgi). The designed EST-SSR primers were synthesized by Sangon laboratory (Shanghai, China).

### EST-SSR amplification and validation

A total of 20 individuals from four populations (Supplementary Table S1) of *R. rex* were selected for polymorphism analyses using the obtained EST-SSRs. Whole genomic DNA of each individual was extracted from the dried leaves using the modified CTAB method (Doyle, 1991). PCR reactions were referenced and modified into a total reaction volume of 20 μL containing 20 ng of DNA, 2 μL of 10 × PCR buffer, 1.6 μL of dNTPs (10 mM), 0.4 μL of each primer, 0.3 μL of Taq DNA polymerase (5 U/μL; Takara, Shiga, Japan), and 14.3 μL of double-distilled water. For each reaction, the following conditions were used: initial 4 min of denaturation at 94°C, followed by 35 cycles of 40 s at 94°C, 25 s of annealing at Tm under different primers, 30 s of extension at 72°C, and a final extension for 10 min at 72°C. All purified PCR products were bidirectionally sequenced by Sangon in a standard sequencing protocol. To test the availability of novel markers, we calculated the number of alleles, *Fst, Ho*, and *He* of all microsatellite loci using GenAlEx version 6.3 (Peakall and Smouse, 2006) and the PIC values by PIC_CALC version 0.6 (Botstein et al., 1980). Hardy-Weinberg equilibrium (HWE) for each locus was calculated by Genepop version 4.1.4 (Yeh et al., 1997). The unweighted pair group mean analysis was estimated by TFPGA version 1.3 (Miller, 1997) with 5,000 permutations. Principal coordinates analysis (PCA) was visualized R software by ape package (Paradis et al., 2004).

### Cross-species amplification and transferability analysis

Eighteen related species of the genus *Rhododendron*, including seven species from subgen. *Hymenanthes* (*R. davidii, R. annae, R. denudatum, R. williamsianum, R. griersonianum, R. protistum* var. *giganteum*, and *R. delavayi*), three species from subgen. *Rhododendron* (*R. ciliicalyx, R. siderophyllum*, and *R. yunnanense*), two species from subgen. *Tsutsusi* (*R. simsii* and *R. pulchrum*), two species from subgen. *Azaleastrum* (*R. hancockii* and *R. latoucheae*), three species from subgen. *Pseudorhodorastrum* (*R. fuyuanense, R. spinuliferum*, and *R. spiciferum*), and one species belonging to the subgen. *Pentanthera* (*R. molle*), were chosen to evaluate the transferability of these newly developed EST-SSR markers. The genomic DNA extraction and PCR amplification were performed as previously described.

## Results

### Illumina sequencing and *de novo* assembly

A total of 56,989,279 paired-end raw reads that contain the adapter-primer sequences, low-quality sequences, and empty reads were generated to construct the cDNA library in Illumina NGS runs for *R. rex*. After a rigorous quality check and data filtering, 55,612,495 high-quality clean reads were obtained with 98.22% Q20 and 94.69% Q30 bases. The clean reads had a total nucleotide number of 8,238,889,450 nt, and the GC percentages for the clean reads were 49.77% (Table 1). Additionally, the high-quality reads were deposited into the U.S. National Center for Biotechnology Information (NCBI) sequence read archive (SRA) database (Raw Illumina reads: NCBI SRP108011).

**Table 1:** Summary of the analysis of *de novo* assembled EST-SSRs for *R. rex*.

**Category**	**Items**	**Number**
Raw reads	Total raw read	56989279
Clean reads	Total clean reads	55612495
	Total clean nucleotides (nt)	8238889450
	Q20 percentage	98.22%
	Q30 percentage	94.69%
	GC percentage	4%
Unigenes	Total sequence number	164242
	Total sequence base	86512813
	Largest	61022
	Smallest	201
	Average	526.74
	N50(bp)	752
	N90 (bp)	238
EST-SSR	Total number of examined sequences	164242
	Total size of examined sequences (bp)	86512813
	Total number of identified SSRs	15314
	Number of SSR-containing sequences	12188
	Number of sequences containing more than one SSR	2483
	Number of SSRs present in compound formation	1045

**Figure 1:**
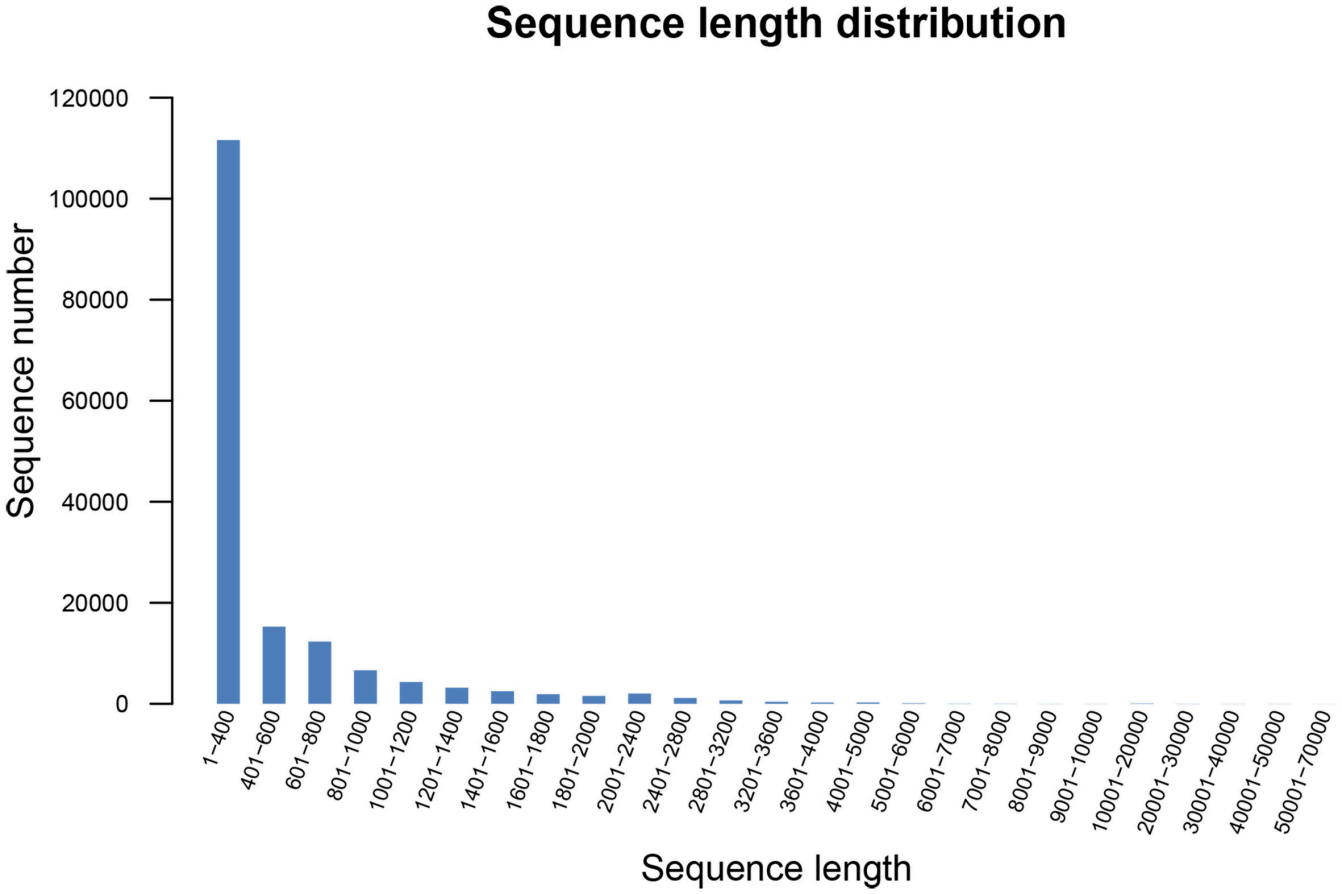
Length distribution of all unigenes in *R. rex*. The x-axis represents the size of all unigenes, and the y-axis represents the number of all unigenes with a certain length.

The total number of unigenes with paired-end reads was 164,242, and the total length of the unigenes was 86,512,813 bp, with an average length of 526.74 bp and the N50 and N90 value of 752 and 238 bp, respectively. In the 164,242 unigenes, the length of 111,599 unigenes (67.95%) ranged from 1 to 400 bp; the length of 34,206 unigenes (20.83%) ranged from 401 to 1,000 bp; the length of 17,853 unigenes (10.88%) ranged from 1,001 to 4,000 bp; and 584 unigenes (0.36%) had a length of more than 4,000 bp. The length distribution of unigenes is shown in Figure 1.

### Functional annotation of unigenes

**Figure 2:**
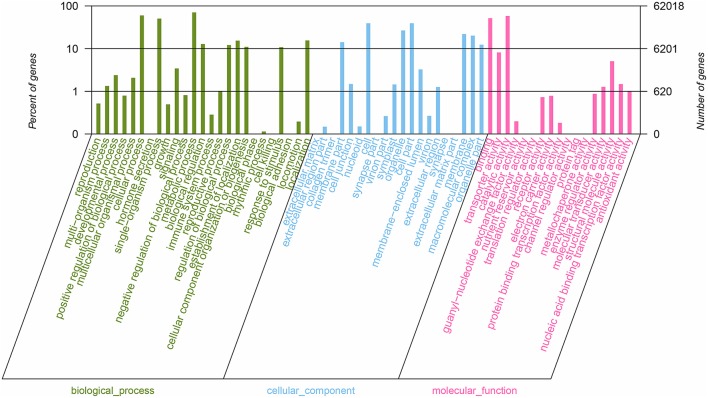
Summary of GO analysis of the unigene sequences of *R. rex*. The y-axis on the right indicates the number of genes in a category. The y-axis on the left indicates the percentage of a specific category of genes in that main category.

**Figure 3:**
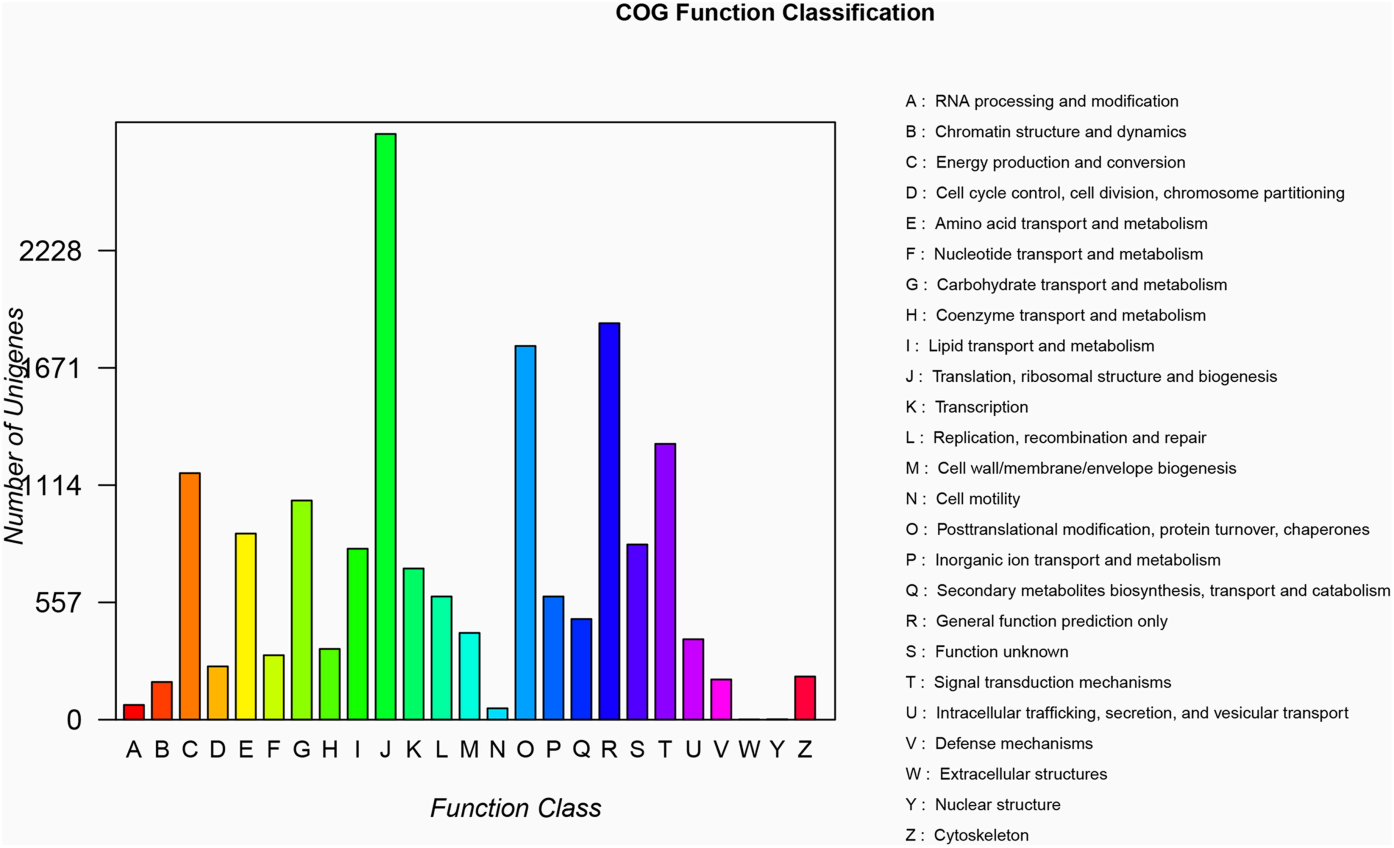
COG analysis of the unigene sequences of *R. rex*. The y-axis indicates the number of unigenes in a specific functional cluster. The x-axis indicates the function class.

**Figure 4:**
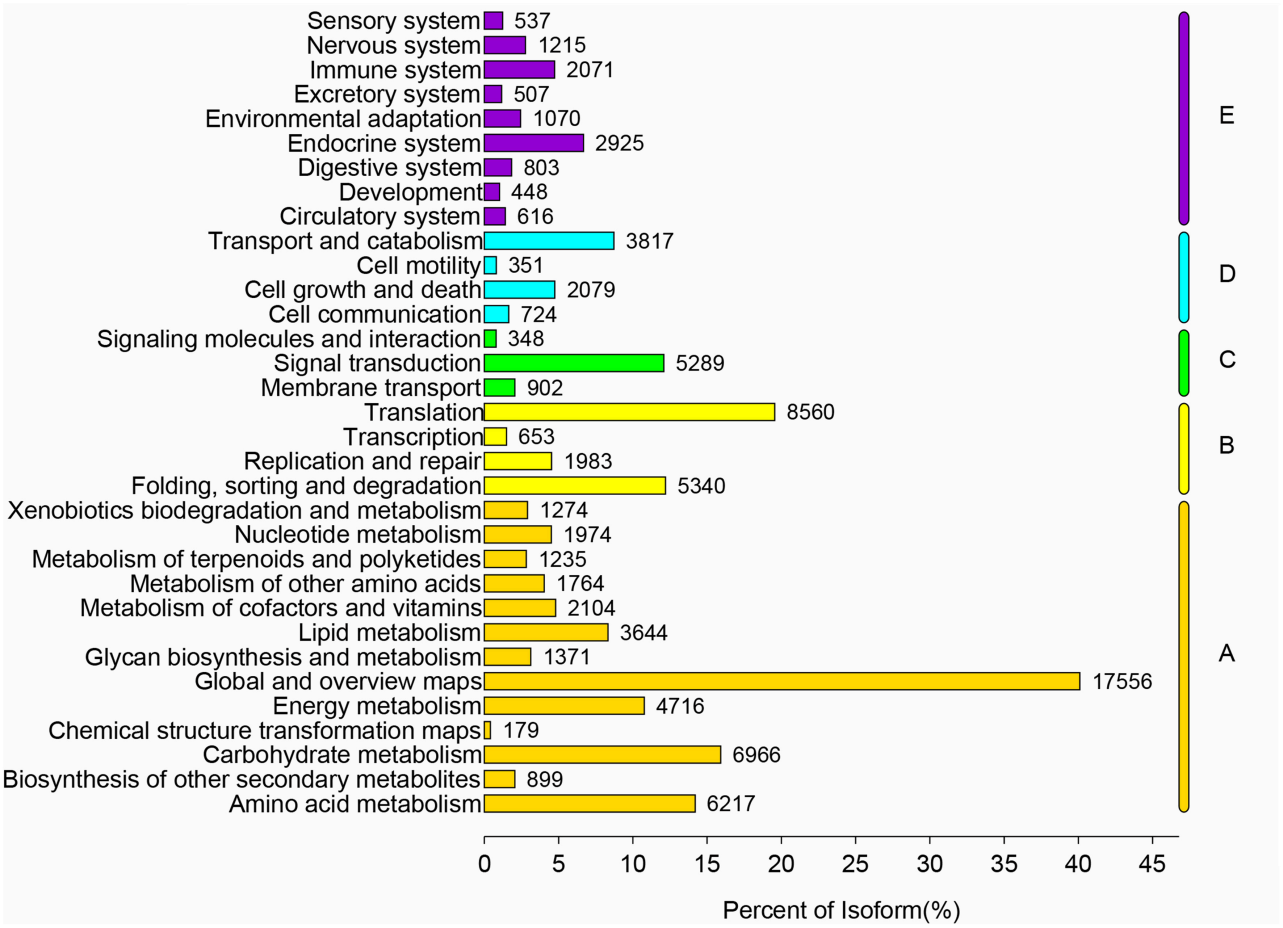
KEGG metabolic pathway of *R. rex*. The y-axis is the name of the KEGG metabolic pathway, and the x-axis is the ratio of the number of genes and the number of genes to the number of genes.

All nucleotide sequences were obtained by splicing, and Blast algorithm (*E* < 1E-5) were used to compare with Nr, String, Swissprot, and KEGG database to obtain similarity sequence and the corresponding annotation information. Of the 164, 242 unigenes, 115,089 unigenes were successfully annotated in the Nr, Pfam, Swiss-Prot, KEGG, COG, and GO databases, respectively (Supplementary Table S2, S3). 97,839 (59.57%) unigenes were aligned to the Nr database, and 27,565 unigenes (28.17%) of the control sequences had significant homology with the entries in the Nr database (<1E-5), and nearly 62,992 unigenes (64.38%) of the sequences showed more than 80% similarity (Supplementary Figure 1). A total of 62,018 (37.76%) unigenes were assigned to the GO annotations, and these terms could be grouped into biological process (168,696, 46.61%), cellular component (113,005, 31.22%), and molecular function (80,246, 22,17%) of the three main categories to annotations (Figure 2). 17,247 unique sequences were assigned into the 25 COG categories. Of these classifications, the translation, ribosomal structure, and biogenesis only (2,782, 16.13%) represented the largest group, followed by general function prediction only (1,883, 10.91%) and posttranslational modification, protein turnover, chaperones (1,775, 10.29%) (Figure 3). 63,183 (38.47%) unigenes had significant matches in the KEGG pathway database and were assigned to five main categories, including 389 KEGG pathways. In the total number of genes involved the pathway, in the five main categories, metabolism was the biggest category (49,899; 55.36%), followed by genetic information processing (16,536; 18.35%), organismal systems (10,192; 11.31%), cellular processes (6,971; 7.73%), and environmental information processing (6,539; 7.25%) (Figure 4).

### Frequency and distribution of SSRs in the unigenes

A total of 15,314 potential EST-SSRs were identified from 12,188 unigenes, with 2,483 unigenes containing more than one EST-SSR locus. Of the 15,314 EST-SSRs, 1,045 were compound microsatellites (Table 1). The frequency of SSRs in the *R. rex* unigenes was 9.32%; an average of one EST-SSR was found every 5.65 kb. The types and distributions of 15,314 potential EST-SSRs were investigated. The most abundant type was repeated di-nucleotide (8,366, 54.63%), followed by mono- (3,986, 26.03%), tri- (2,835, 18.51%). Table 2 shows that the six tandem repeats of the EST-SSR (2,533, 16.54%) were the most common, followed by ten tandem repeats (2,272, 14.84%), nine tandem repeats (2,088,13.63%), and eight, seven, and five tandem repeats were (1,962,12.81%), (1,887,12.32%), (1,871,12.21%), whereas the rest of the tandem repeat each accounted for <10% of the EST-SSR. The EST-SSR tandem repeat was 10–478 bp in length, and 18 bp was the most frequently observed length (18.5%).

**Table 2:** Length distribution of the EST-SSRs of *R. rex* based on the number of nucleotide repeat units.

**Number of repeats**	**Mono-**	**Di-**	**Tri-**	**Quad-**	**Penta-**	**Hexa-**	**Total**	**Percentage (%)**
5			1,777	69	18	7	1,871	12.21
6		1,794	715	18		6	2,533	16.54
7		1,566	317	2	2		1,887	12.32
8		1,940	21			1	1,962	12.81
9		2,086	1			1	2,088	13.63
10	1,418	853				1	2,272	14.84
11	787	121	1				909	5.94
12	509	6					515	3.36
13	351						351	2.29
14	259						259	1.69
15	197						197	1.29
16	120						120	0.78
17	108						108	0.71
18	75					2	77	0.5
19	47		1				48	0.31
20	42		1				43	0.28
21	38						38	0.25
22	26						26	0.17
23	8						8	0.05
24	1		1				2	0.01
Total	3,986	8,366	2,835	89	20	18	15,314	
Percentage (%)	26.03	54.63	18.51	0.58	0.13	0.12		

In the di-nucleotide repeats, AG/CT was the most abundant motif (7,632; 49.84%), followed by AC/GT (413; 2.7%), AT/AT (285; 1.86%), and CG/CG (36; 0.24%). In the tri-nucleotide repeats, the most dominant repeat motif was AAG/CTT (628; 4.1%), followed by AGG/CCT (456; 2.98%), AGC/CTG (425; 2.78%), ACC/GGT (412; 2.69%), CCG/CGG (256; 1.67%), ATC/ATG (211; 1.38%), AAC/GTT (211; 1.38%), ACG/CGT (129; 0.84%), AAT/ATT (59; 0.39%), and ACT/AGT (48; 0.31%) (Supplementary Table S4). The 14 repeat motifs mentioned above accounted for 73.16% of the total, whereas the remaining 56 types accounted for 26.84%.

### Development and validation of new EST-SSR markers

Based on the SSR-containing sequence, 100 primer pairs were randomly selected and synthesized and were successfully used for validation of the amplification and assessment of the polymorphism. In the selected 100 primer pairs, 50 were successfully amplified by *R. rex* genomic DNA, and the remaining 50 pairs of primers failed to amplify PCR products at various annealing temperatures. Among the 50 successful primer pairs, 45 produced the expected size of the amplified products, and the other five pairs of primers produced PCR products that were larger or smaller than expected. Using 20 *R. rex* individuals from four populations as PCR templates, the results showed that 36 of the 45 primer pairs were polymorphic, and nine pairs were identified as monomorphic.

The 36 polymorphic EST-SSR markers developed in this study were used to assess the 20 genotype individuals. As presented in Table 3, 15 of 36 loci most significantly deviate from the equilibrium state in HWE test (*P* < 0.0001). A total of 197 alleles were detected. The number of alleles varied from 2 to 11, with a mean of 5.472 alleles per locus. Observed heterozygosity ranged from 0.050 to 0.700 with a mean of 0.323, and expected heterozygosity ranged from 0.045 to 0.698 with a mean of 0.372. The PIC ranged from 0.154 to 0.870, with a mean of 0.482. The *Fst* ranged from 0.092 to 0.736, with a mean of 0.268. The null allele frequency ranged from 0.000 to 0.972, with a mean of 0.263 (Table 3). In additional, the consequence of UPGMA clustering analysis and PCA analysis all showed that a distinctly genetic structure existed between the 4 *R. rex* populations by the 36 novel EST-SSR markers (Supplementary Figures 2, 3).

**Table 3:** Characteristics of the 36 novel EST-SSR markers in *R. rex*.

**Primer**	**Forward primer (5″–3″)**	**Reverse primer (5″–3″)**	**Tm (°C)**	**SSRs**	**Number of alleles**	**Ho**	**He**	**PIC**	**HWE**	**Fst**	**r**
Rho-1	ACCGAGTCACAGCACTCCTT	CACATTCATCCTCCCCAATC	52	(CGC)5	3	0.050	0.165	0.412	0.0003^***^	0.676	0.331
Rho-4	TTGACGAACTGCACCAACTC	TTCAGTCACAAACCTGCATCA	55	(TG)6	3	0.083	0.153	0.166	0.0001^***^	0.323	0.231
Rho-10	GGGAGAGAGAGGTCCTACCG	CTGCCCTTGTTTGACGATTT	60	(GA)7	11	0.433	0.685	0.870	0.0000^***^	0.221	0.265
Rho-11	GAGGACGAGGGTGGTACAAA	AAGCCATGGAGTTGATACGG	60	(GCA)7	3	0.350	0.299	0.338	0.0000^***^	0.222	0.135
Rho-13	AGCCCTTCCTCTGTCTCCTC	TTCGAATGGATCAAATGGGT	58	(CTC)5	4	0.200	0.160	0.208	0.9998*ns*	0.141	0
Rho-14	GAGATTCTCAACCCAACCCA	TCCAAACAGACATCCGATCA	51	(GA)6	5	0.263	0.342	0.490	0.0000^***^	0.390	0.226
Rho-15	CAATCAAGGGGCTACCATGT	CGAAAGTTGGTGGTATCCGT	57	(AGG)6	5	0.700	0.580	0.618	0.1278*ns*	0.134	0.241
Rho-17	AGTGGACAGTGAGGTCACCC	TCGGATGAATTGCGTTGTAA	60	(TGT)5	4	0.588	0.391	0.520	0.8759ns	0.364	0.578
Rho-20	TCCTGGGTCCATAACACACC	GGTCACGTGTCTGAGCGTAA	60	(AC)9	2	0.200	0.120	0.352	0.0325^*^	0.736	0.485
Rho-26	CCTGAATCCATCCTGTCCTG	GCTGAGGGATCACCAGACAT	56	(GA)8	9	0.700	0.583	0.798	0.0024^*^	0.303	0.176
Rho-27	GGGGTAATACCGGAGGGTAA	GTTCCCTGAAGACATGGTGG	56	(GA)7	3	0.200	0.295	0.406	0.0012^*^	0.312	0.391
Rho-30	GGAAGTTTCGGCAGCAGTAG	CCTTCTCCCAACTCCCTTTC	56	(GGT)5	7	0.650	0.498	0.564	0.2730ns	0.169	0.11
Rho-33	CCACCCTTCCCTTATCCTTG	GAGAAGTGTGGCTTTGAGGG	56	(CT)7	9	0.450	0.571	0.737	0.5345ns	0.237	0.224
Rho-34	AGTGGCCTTAGGGGAAAGAA	CAACCCTTACCCACCACATC	56	(GGC)5	2	0.050	0.045	0.053	1.0000ns	0.077	0.972
Rho-35	TGAATCCACCACAACAAGGA	ACTCCCCTCTCGGAAATTGT	55	(TA)8	4	0.333	0.334	0.473	0.1908ns	0.392	0.161
Rho-37	CATGGAGAAGACCCACTGGT	ACCCACGCATTAACTTCAGG	56	(CT)8	9	0.450	0.698	0.820	0.0000^***^	0.169	0.324
Rho-39	GTGGCTCAAAATACAGGGGA	CAGATGAAGGCGATGTGAGA	58	(GCT)5	5	0.150	0.355	0.368	0.0129^*^	0.123	0.209
Rho-45	CTATGGCGGGCCTATCTGT	CATACGAAGGACGAGGTGGT	60	(TCC)6	8	0.400	0.670	0.700	0.0000^***^	0.092	0.238
Rho-48	CTGCGTCTTTGGGTTTCTTC	CAATCCAACCCACCATTTTC	55	(GAG)5	5	0.113	0.313	0.333	0.0000^***^	0.167	0.238
Rho-51	AAATATGTTCACCCCCACCA	GCCTGGACTGTTGGAATGTT	58	(CAC)5	5	0.163	0.262	0.271	0.0058^*^	0.097	0.175
Rho-53	AACCAGTACAGGACGCCAAG	CTCCCGAGAAGATCAAGCAG	56	(GCG)5	3	0.050	0.345	0.345	0.0001^**^	0.161	0.481
Rho-54	GAAATACGGAAACGGACGAA	CCTCTCTCTCTCCGCACATT	52	(TGTA)5	3	0.392	0.332	0.517	0.1424ns	0.435	0.26
Rho-56	CCTCCTCTCGCATCATTCTC	CACTGCCATCTCTCACTCCA	56	(TCG)5	4	0.213	0.207	0.237	0.0001^**^	0.169	0.11
Rho-59	AAACCTCTCGCTCTCTTCCC	GGTGTCGGTCTTCATGGTTT	56	(CT)7	9	0.725	0.623	0.821	0.1762ns	0.260	0.096
Rho-62	ATATGTTGCGCGGGAGAAT	TCTCGAAGGCAAAACAGCTT	54	(CT)6	5	0.250	0.345	0.365	0.0000^***^	0.101	0.195
Rho-67	GGTGGATCAGAAGGGACTGA	ACATGAAGATCATGGGCGAT	55	(CTT)5	8	0.375	0.501	0.579	0.0000^***^	0.169	0.196
Rho-69	CGAATCCTCCATCAAAGCAT	ATGCAAAACTGTGACCTCCC	57	(TTC)5	5	0.100	0.355	0.651	0.0000^***^	0.459	0.425
Rho-70	GGCTGTGAGGGAGTCAAAGA	TCTCCATTGTCGAAACCTCC	56	(GA)6	7	0.450	0.383	0.410	0.0246^*^	0.162	0
Rho-74	ATAACGCGCAAACTAGCGTT	ATGAGGAGGAGCGCACTTTA	58	(CTG)5	6	0.400	0.455	0.671	0.0001^**^	0.366	0.502
Rho-75	GTAAATGGGCCCGTATTCCT	CTCCATTGAGAAACCCTCCA	54	(TTG)6	4	0.150	0.120	0.154	0.9999ns	0.158	0
Rho-79	TGGTTCTGTTCTCTGGCCTC	TTCCAGGATAGTGCTCCTGC	58	(TC)7	9	0.400	0.415	0.684	0.0000^***^	0.428	0.22
Rho-81	ATGGATCGTTCTGGACGAAG	AAGGCCACTAGAAGAAGCCC	60	(CT)8	10	0.613	0.601	0.801	0.0011^**^	0.275	0.232
Rho-84	TGACGGACTTGTGCTGGATA	GAGAAAAGGGAAGAAGGACACA	58	(TC)7	2	0.050	0.310	0.372	0.0002^***^	0.373	0.642
Rho-93	TGCAGAGTAAAACCCTGCTTG	TAAAGTTGAGGCGGCAAAGT	58	(CA)7	3	0.050	0.085	0.099	0.0000^***^	0.117	0.153
Rho-95	GGGGTAGGGGGATACTTTGA	GTCGACGACTTTGGTCCAGT	55	(GCT)6	8	0.450	0.395	0.509	0.0001^***^	0.249	0.084
Rho-97	TTTGCGGTGGTGTCTGAATA	AAATCCAATGATCCATCCCA	55	(GA)7	5	0.425	0.404	0.631	0.0016^**^	0.412	0.159
Mean					5.472	0.323	0.372	0.482		0.268	0.263

ns, non-significance;

* p < 0.05, significant difference;

** p < 0.01, most significant difference;

*** *P < 0.001, most significant difference; r, null allele frequency*.

### Transferability of the newly developed EST-SSR markers

A total of 36 pairs of primers were evaluated for transferability in 18 species (including seven species of subgen. *Hymenanthes*, three species of subgen. *Rhododendron*, two species of subgen. *Azaleastrum*, two species of subgen. *Tsutsusi*, three species of subgen. *Pseudorhodorastrum*, and one species of subgen. *Pentanthera*) of the genus *Rhododendron*. Nine primer pairs successfully amplified all of the templates. The transferability ratios ranged from 58.33 to 83.33% (subgen. *Hymenanthes*: 63.89%; subgen. *Rhododendron*: 66.67%; subgen. *Azaleastrum*: 83.33%; subgen. *Tsutsusi*: 63.89%; subgen. *Pseudorhodorastrum*: 58.33%; and subgen. *Pentanthera*: 72.22%).

## Discussion

### Characterization of the *R. rex* transcriptome

NGS has been increasingly used to analyze transcriptome sequencing and assembly in many plants because of its high efficiency, speed, accuracy, and low cost (Chen H. et al., 2015; An et al., 2016; Xing et al., 2017). SSR markers, due to their high polymorphism, high repeatability, and codominant inheritance, have been widely used to construct DNA fingerprints, genetic diversity analysis, gene mapping, and molecular marker-assisted breeding (Morgante et al., 1994; Agarwal et al., 2008; Luo et al., 2010). Deep transcriptome sequencing, which generates massive data, provides comprehensive information and a good resource for development of SSRs and gene discovery in many organisms (Zheng et al., 2013; Huang et al., 2014; Chen H. et al., 2015). Thus, markers based on transcriptome sequences have been successfully used for the detection of functional variation and gene-associated genetic analysis both in model and non-model species (Trick et al., 2009; Zheng et al., 2013; Yan et al., 2015). However, reports on transcriptome sequencing are few and available EST sequences of *Rhododendron* are also limited. In the present study, we first reported the transcriptome *of R. rex* by utilizing transcriptome sequences generated via NGS technologies. A total of 56,989,279 paired-end raw reads were generated and 55,612,495 high-quality clean reads were obtained with 98.22% Q20 level, which ensures the quality of sequencing and is consistent with the results reported in *Neolitsea sericea* (Chen L. Y. et al., 2015) and *Elymus sibiricus L*. (Zhou et al., 2016). Compared with *Quercus austrocochinchinensis* (An et al., 2016) and *R. latoucheae*. (Xing et al., 2017), the mean N50 sizes (752 bp) of unigenes generated in this research were shorter. However, 164,242 unigenes were assembled from the *R. rex* transcriptome with an average length of 526.74 bp, which was longer than that reported in previous transcriptome studies of tea (402 bp; Tan et al., 2013), *Hevea brasiliensis* (485 bp; Li et al., 2012), and *Rosa roxburghii* (343 bp; Yan et al., 2015) but shorter than *N. sericea* (733 bp; Chen L. Y. et al., 2015), *Cocos nucifera* (752 bp; Xia et al., 2014), and *Juglans mandshurica* (752 bp; Hu et al., 2016). This result may be related to the difference between the assembler and the parameters and the differences in the nature of species. Overall, the large number of transcriptome sequences generated in the present study will be valuable for exploring the molecular mechanisms and for gene function.

The assembled unigenes of *R. rex* were successfully annotated to the known public databases Nr, Pfam, Swiss-Prot, KEGG, COG, and GO. These relatively complete annotations can provide valuable information for the future genetic diversity and evolutionary adaptation analysis of *R. rex*. In addition, some genes cannot match any functional annotations, possibly due to short sequence length discrepancies or limited annotation of the genus *Rhododendron* and related species in the current database (Xing et al., 2017). However, these failed annotations were still important resources for *R. rex* conservation or utilization. A large number of genomes are assigned to a wide range of GO categories, and COG classifications indicated that assembled unigenes represent a wide variety of transcripts in the *R. rex* genome. Of the three GO categories, metabolic process and cell under the biological process and cellular component were the largest group in our study, which were consistent with the report from Li et al. (2012). Meanwhile, we also found that some sequences in the GO database were annotated for the functions of response to temperature stimulus (GO: 0009266), photosynthesis (GO: 0015979), and osmotic stress (GO: 0006970), such as catalase (K03781) has been identified involved in plant cell growth as well as cold tolerance in other plants (Mutlu et al., 2013; Xu et al., 2013). Wei et al. (2005) also proposed that osmotic regulation, photoinhibition tolerance, and photosynthesis adjustment are important components for cold adaptation in *Rhododendron*. Thus, these sequences may represent a category of genes for cold adaptation and could be used to reveal evolutionary adaptation of cold tolerance mechanisms in a future study. In the COG classification, the first and second classifications found in our work were the translation, ribosomal structure, and biogenesis only and general function prediction only, which was similar to Yan et al. (2015) but was different from the report by Li et al. (2012). Moreover, 63,183 (38.47%) unigenes had significant matches in the database and were assigned to five main categories, including 389 KEGG pathways. These results not only revealed the active metabolic processes in *R. rex* but also mean that various metabolites are synthesized in this species.

### Frequency and distribution of EST-SSRs

The EST-SSR markers are important in the research of genetic diversity and population structure assessment in the species conservation, development of genetic maps, genomics comparison, and marker-assisted selection breeding (Chen L. Y. et al., 2015; Xing et al., 2017). In this study, a total of 15,314 potential EST-SSRs were identified from 12,188 unigenes, significantly improving the availability of SSR resources for marker development in this endangered and endemic species. Di-nucleotide (54.63%) repeats were the most abundant type, which was consistent with previous studies (Triwitayakorn et al., 2011; Yan et al., 2015; Xing et al., 2017), followed by mono- (26.03%) and tri-nucleotide (18.51%) repeats (Table 2). This result was consistent with previous findings that di-nucleotide repeats are the most common SSR repeat type in dicotyledonous plants (Kumpatla and Mukhopadhyay, 2005; Chen L. Y. et al., 2015). Studies have shown that the advantage of the di-nucleotide repeat sequence may be due to the over-expression of UTRs compared with open reading frames (Kumpatla and Mukhopadhyay, 2005; Qiu et al., 2010). In addition, the most abundant di-nucleotide repeats were AG/TC (49.84%). The AG/CT motif can represent UCU, and CUC codons in an mRNA population and translates into the amino acids Ala, and Leu which present in proteins at higher frequencies than other amino acids (Chen L. Y. et al., 2015). Thus, AG/CT motifs are present at high frequencies in EST collections of plants (Morgante et al., 2002; Chen L. Y. et al., 2015). CG/CG (0.24%) was the least abundant motif in our result. It might be due to methylation of cytosine, which would inhibit the transcription of some plants (Chen L. Y. et al., 2015; Xing et al., 2017). In the tri-nucleotide repeats, the most dominant repeat motif was AAG/CTT (4.1%). Previous studies of Arabidopsis, soybean, *Cucumis sativus, Ricinus communis, Sesamum indicum, N. sericea* also showed that the tri-nucleotide AAG motif may be significantly prominent in dicotyledonous plants (Morgante et al., 2002; Cavagnaro et al., 2010; Qiu et al., 2010; Wei et al., 2011; Chen L. Y. et al., 2015). The frequency of SSRs in the *R. rex* unigenes (1/5.65 kb) is much higher than those obtained in *Elymus sibiricus* (1/6.59 kb) (Zhou et al., 2016) and tree peony (1/9.24 kb) (Wu et al., 2014) but lower than the frequency of *Camellia sinensis* (1/4.99 kb) (Wu et al., 2013), *R. latoucheae* (1/2.87 kb) (Xing et al., 2017), and *N. sericea* (1/3.8 kb) (Chen L. Y. et al., 2015). The frequency is speculated to depend to some extent on the species differences that are also attributed to the SSR search criteria, database size, and the database-mining tools of different studies (Varshney et al., 2005; Biswas et al., 2012).

### Validation and transferability of EST-SSR makers

In the selected 100 primer pairs that were randomly selected for PCR validation, 50 were successfully amplified by *R. rex* genomic DNA, and the remaining 50 pairs of primers failed to amplify PCR products at various annealing temperatures. 36 (36%) were polymorphic among the four *R. rex* populations tested, and the remaining 64 pairs of primers failed to amplify PCR products at various annealing temperatures or produced PCR products that were larger or smaller than expected. This PCR success rate was higher than the rates reported for *N. sericea* (16.03%) (Chen L. Y. et al., 2015), *E. sibiricus* (22.40%) (Zhou et al., 2016), and *J. mandshurica* (30.8%) (Hu et al., 2016), and *Chrysanthemum nankingense* (20%) (Wang et al., 2013) but lower than the rates reported for tree peony (39.90%) (Wu et al., 2014). Thus, the polymorphic ratio of EST-SSRs in this study was at a relatively high level. The level of polymorphism may be influenced by the amount of material used and the different geographic origin used in the study (Wu et al., 2014; Zhou et al., 2016).

Using the 36 polymorphic EST-SSR makers, 197 alleles were detected among the 20 *R. rex* individuals from four populations. The number of alleles per locus ranged from 2 to 11 with a mean of 5.47 alleles, expected heterozygosity (*He*) averaging 0.372, and observed heterozygosity (*Ho*) averaging 0.323, which is lower than that of the genomic SSRs in *Lobelia deckenii* (*Ho* = 0.762, *He* = 0.563) (Mi et al., 2015) and *Elymus sibiricus L*.(*Ho* = 0.49, *He* = 0.59) (Zhou et al., 2016). The PIC value of each EST-SSR reflects the allelic diversity and frequency of the sampled individuals and was used to evaluate the level of information (Botstein et al., 1980; Wu et al., 2014). In our study, PIC values ranged from 0.154 to 0.870, averaging 0.482, which present a moderate level. Thus, it indicated that the newly developed EST-SSR markers were an informative and effective tool for the genetic analysis, and evolutionary adaptation among a wide range of diverse *Rhododendron* species at the species level. However, since previous studies suggest that 20–30 individuals should be sampled in SSRs studies (Hale et al., 2012), we suggest that the species' genetic diversity and structure requires further investigation by expanding the sampling scale.

Previous studies have shown that the EST-SSR located in the transcribed region has a high degree of transferability (10–90%) among the related species and established the range of variation of EST-SSR across species (Ellis and Burke, 2007; Wu et al., 2014). In the present study, the 36 polymorphic EST-SSR markers developed were used to evaluate for transferability in 18 species from six subgen. in the family Ericaceae. The transferability ratios ranged from 58.33 to 83.33%, which is higher than that obtained in bottle gourd (4–41%) (Xu et al., 2011), *Elymus* (49.11%) (Zhou et al., 2016), and *Cucumis* (12.7%) (Fernandez-Silva et al., 2008). Thus, the high transferability across the part of this genus in the present study will provide valuable sequence resources for the molecular marker development for *Rhododendron* species.

## Conclusion

We used Illumina HiSeq4000 sequencing as well as *de novo* transcriptome assembly and annotation of *R. rex*. We also successfully identified and developed a set of EST-SSR markers in the present study. A total of 164, 242 unigenes with a mean length of 526.74 bp from *R. rex* was generated. A total of 115,089 unigenes were successfully annotated into the Nr (97,839: 59.57%), Pfam (37,981: 23.13%), Swiss-Prot (60,600: 36.9%), KEGG (63,183: 38.47%), COG (17,247: 10.50%), and GO (62,018: 37.76%) databases. Based on these unigene sequences, a total of 15,314 potential EST-SSRs were identified and characterized. A total of 100 primer pairs were randomly selected and synthesized and used for assessment of the polymorphism, thirty-six primer pairs were polymorphic and revealed polymorphism among 20 individuals from four *R. rex* populations. The newly developed EST-SSR markers have high transferability (58.33–83.33%) in the present study. These results would not only provide an important resource for future genetic and genomic studies of *R. rex* but also represent a powerful molecular tool for evolutionary adaptation and genetic relationship analysis in other *Rhododendron* species.

## Author contributions

SS, YZ, XZ, and YW initiated and designed the research. SS obtained funding for this study. YZ, XZ, and SS collected the materials and performed the experiments, SS, YZ, and XZ wrote and revised the paper. All authors read and approved the manuscript.

### Conflict of interest statement

The authors declare that the research was conducted in the absence of any commercial or financial relationships that could be construed as a potential conflict of interest.
