# Supplementary material for: De Novo Assembly of Transcriptome and Development of Novel EST-SSR Markers in Rhododendron rex Lévl. through Illumina Sequencing
Source: Front Plant Sci. 2017 Sep 26;8:1664. doi: 10.3389/fpls.2017.01664 (PMC5622969; doi:10.3389/fpls.2017.01664)
Supplement: Supplementary file 1 [file Table1.DOC]

Supplementary Material

**Characterization of transcriptome and development of novel EST-SSR markers in *Rhododendron rex* Lévl. through Illumina sequencing**

**Authors:** Yue Zhang, Xue Zhang, Yue-Hua Wang, Shi-Kang Shen*

School of Life Sciences, Yunnan University, Kunming No. 2 Green lake North road Kunming, Yunnan, 650091, China.

***Correspondence author:** Shi-Kang Shen

**Supplementary Table S1**  *R. rex* accessions used for marker validation

| No. | Source | Longitude (E) | Latitude (N) | Altitude (m) |
| --- | --- | --- | --- | --- |
| Pop1 | Luquan,Yunnan | 102°49′56.8″ | 26°04′07.3″ | 3250 |
| Pop2 | Chuxiong,Yunnan | 101°3′11″ | 26°3′26″ | 2950 |
| Pop3 | Jingdong,Yunnan | 100°41′47″ | 24°28′57″ | 2660 |
| Pop4 | Jingdong,Yunnan | 100°38′15″ | 24°24′31″ | 2750 |
