# Supplementary material for: De Novo Assembly of Transcriptome and Development of Novel EST-SSR Markers in Rhododendron rex Lévl. through Illumina Sequencing
Source: Front Plant Sci. 2017 Sep 26;8:1664. doi: 10.3389/fpls.2017.01664 (PMC5622969; doi:10.3389/fpls.2017.01664)
Supplement: Supplementary file 2 [file Table2.DOC]

Supplementary Material

**Characterization of transcriptome and development of novel EST-SSR markers in *Rhododendron rex* Lévl. through Illumina sequencing**

**Authors:** Yue Zhang, Xue Zhang, Yue-Hua Wang, Shi-Kang Shen*

School of Life Sciences, Yunnan University, Kunming No. 2 Green lake North road Kunming, Yunnan, 650091, China.

***Correspondence author:** Shi-Kang Shen

**Supplementary Table S2** Functional annotation of the R.rex transcriptome.

| Category | Number | Percentage(%) |
| --- | --- | --- |
| Pfam | 37981 | 23.13 |
| Nr annotation | 97839 | 59.57 |
| Swiss-Prot annotation | 60600 | 36.9 |
| KEGG annotation | 63183 | 38.47 |
| COG annotation | 26808 | 16.32 |
| GO annotation | 62018 | 37.76 |
