# Supplementary material for: De Novo Assembly of Transcriptome and Development of Novel EST-SSR Markers in Rhododendron rex Lévl. through Illumina Sequencing
Source: Front Plant Sci. 2017 Sep 26;8:1664. doi: 10.3389/fpls.2017.01664 (PMC5622969; doi:10.3389/fpls.2017.01664)
Supplement: Supplementary file 4 [file Table4.DOC]

Supplementary Material

**Characterization of transcriptome and development of novel EST-SSR markers in *Rhododendron rex* Lévl. through Illumina sequencing**

**Authors:** Yue Zhang, Xue Zhang, Yue-Hua Wang, Shi-Kang Shen*

School of Life Sciences, Yunnan University, Kunming No. 2 Green lake North road Kunming, Yunnan, 650091, China.

***Correspondence author:** Shi-Kang Shen

**Supplementary Table S4**  Detailed information of EST-SSRs based on the number of nucleotide repeat unit in *R. rex*

| Repeats | 5 | 6 | 7 | 8 | 9 | 10 | 11 | 12 | 13 | 14 | ≥15 | total | Percentage  (%) |
| --- | --- | --- | --- | --- | --- | --- | --- | --- | --- | --- | --- | --- | --- |
| A/T | - | - | - | - | - | 1381 | 759 | 500 | 339 | 255 | 624 | 3858 | 25.19 |
| C/G | - | - | - | - | - | 37 | 28 | 9 | 12 | 4 | 38 | 128 | 0.84 |
| AC/GT | - | 160 | 116 | 56 | 38 | 30 | 12 | 1 | - | - |  | 413 | 02.70 |
| AG/CT | - | 1504 | 1375 | 1836 | 2019 | 804 | 90 | 4 | - | - |  | 7632 | 49.84 |
| AT/AT | - | 100 | 69 | 48 | 29 | 19 | 19 | 1 | - | - |  | 285 | 1.86 |
| CG/CG | - | 30 | 6 | - | - | - | - | - | - | - |  | 36 | 0.24 |
| AAC/GTT | 120 | 60 | 27 | 4 | - | - | - | - | - | - |  | 211 | 1.38 |
| AAG/CTT | 372 | 170 | 83 | 2 | - | - | 1 | - | - | - |  | 628 | 4.10 |
| AAT/ATT | 38 | 7 | 11 | 3 | - | - | - | - | - | - |  | 59 | 0.39 |
| ACC/GGT | 256 | 104 | 51 | - | - | - | - | - | - | - | 1 | 412 | 2.69 |
| ACG/CGT | 88 | 30 | 10 | 1 | - | - | - | - | - | - |  | 129 | 0.84 |
| ACT/AGT | 28 | 12 | 7 | 1 | - | - | - | - | - | - |  | 48 | 0.31 |
| AGC/CTG | 263 | 114 | 46 | 2 | - | - | - | - | - | - |  | 425 | 2.78 |
| AGG/CCT | 305 | 110 | 38 | 2 | - | - | - | - | - | - | 1 | 456 | 2.98 |
| ATC/ATG | 124 | 48 | 35 | 3 | - | - | - | - | - | - | 1 | 211 | 1.38 |
| CCG/CGG | 183 | 60 | 9 | 3 | 1 | - | - | - | - | - |  | 256 | 1.67 |
| AAAC/GTTT | 6 | 1 | - | - | - | - | - | - | - | - |  | 7 | 0.05 |
| AAAG/CTTT | 11 | 2 | 1 | - | - | - | - | - | - | - |  | 14 | 0.09 |
| AAAT/ATTT | 7 | - | - | - | - | - | - | - | - | - |  | 7 | 0.05 |
| AACC/GGTT | 3 | 1 | 1 | - | - | - | - | - | - | - |  | 5 | 0.03 |
| AACT/AGTT | 1 | - | - | - | - | - | - | - | - | - |  | 1 | 0.01 |
| AAGG/CCTT | 4 | 2 | - | - | - | - | - | - | - | - |  | 6 | 0.04 |
| AATC/ATTG | 4 | 2 | - | - | - | - | - | - | - | - |  | 6 | 0.04 |
| ACAG/CTGT | 3 | 2 | - | - | - | - | - | - | - | - |  | 5 | 0.03 |
| ACAT/ATGT | 8 | - | - | - | - | - | - | - | - | - |  | 8 | 0.05 |
| ACCG/CGGT | 1 | - | - | - | - | - | - | - | - | - |  | 1 | 0.01 |
| ACGG/CCGT | - | 1 | - | - | - | - | - | - | - | - |  | 1 | 0.01 |
| ACTC/AGTG | 3 | - | - | - | - | - | - | - | - | - |  | 3 | 0.02 |
| AGAT/ATCT | 4 | 1 | - | - | - | - | - | - | - | - |  | 5 | 0.03 |
| AGCC/CTGG | 2 | 1 | - | - | - | - | - | - | - | - |  | 3 | 0.02 |
| AGCG/CGCT | 1 | - | - | - | - | - | - | - | - | - |  | 1 | 0.01 |
| AGCT/AGCT | 1 | - | - | - | - | - | - | - | - | - |  | 1 | 0.01 |
| AGGC/CCTG | - | 2 | - | - | - | - | - | - | - | - |  | 2 | 0.01 |
| AGGG/CCCT | 8 | 3 | - | - | - | - | - | - | - | - |  | 11 | 0.07 |
| ATCG/ATCG | 2 | - | - | - | - | - | - | - | - | - |  | 2 | 0.01 |
| AAAAT/ATTTT | 1 | - | - | - | - | - | - | - | - | - |  | 1 | 0.01 |
| AAACC/GGTTT | 1 | - | - | - | - | - | - | - | - | - |  | 1 | 0.01 |
| AAACT/AGTTT | 1 | - | - | - | - | - | - | - | - | - |  | 1 | 0.01 |
| AAGAG/CTCTT | 3 | - | - | - | - | - | - | - | - | - |  | 3 | 0.02 |
| AAGAT/ATCTT | 1 | - | - | - | - | - | - | - | - | - |  | 1 | 0.01 |
| AAGGC/CCTTG | - | - | 1 | - | - | - | - | - | - | - |  | 1 | 0.01 |
| AAGGG/CCCTT | 1 | - | - | - | - | - | - | - | - | - |  | 1 | 0.01 |
| ACCAT/ATGGT | 1 | - | - | - | - | - | - | - | - | - |  | 1 | 0.01 |
| ACTCC/AGTGG | 1 | - | - | - | - | - | - | - | - | - |  | 1 | 0.01 |
| ACTCG/AGTCG | 1 | - | - | - | - | - | - | - | - | - |  | 1 | 0.01 |
| ACTCT/AGAGT | 1 | - | - | - | - | - | - | - | - | - |  | 1 | 0.01 |
| ACTGC/AGTGC | 1 | - | - | - | - | - | - | - | - | - |  | 1 | 0.01 |
| AGAGG/CCTCT | 2 | - | - | - | - | - | - | - | - | - |  | 2 | 0.01 |
| AGATC/ATCTG | 1 | - | - | - | - | - | - | - | - | - |  | 1 | 0.01 |
| AGATG/ATCTC | 1 | - | - | - | - | - | - | - | - | - |  | 1 | 0.01 |
| AGCTC/AGCTG | 1 | - | - | - | - | - | - | - | - | - |  | 1 | 0.01 |
| AGGAT/ATCCT | - | - | 1 | - | - | - | - | - | - | - |  | 1 | 0.01 |
| AAAAAG/CTTTTT | - | - | - | - | 1 | - | - | - | - | - |  | 1 | 0.01 |
| AAAGGG/CCCTTT | 1 | - | - | - | - | - | - | - | - | - |  | 1 | 0.01 |
| AACACG/CGTGTT | - | 1 | - | - | - | - | - | - | - | - |  | 1 | 0.01 |
| AACAGC/CTGTTG | - | - | - | 1 | - | - | - | - | - | - |  | 1 | 0.01 |
| AACCAC/GGTTGT | 1 | - | - | - | - | - | - | - | - | - |  | 1 | 0.01 |
| AACGAC/CGTTGT | - | - | - | - | - | - | - | - | - | - | 1 | 1 | 0.01 |
| AACTCC/AGTTGG | - | 1 | - | - | - | - | - | - | - | - |  | 1 | 0.01 |
| AAGAGG/CCTCTT | - | 1 | - | - | - | - | - | - | - | - |  | 1 | 0.01 |
| AAGATG/ATCTTC | - | - | - | - | - | - | - | - | - | - |  | 1 | 0.01 |
| AAGTGG/ACTTCC | - | 1 | - | - | - | - | - | - | - | - |  | 1 | 0.01 |
| ACAGGC/CCTGTG | 1 | - | - | - | - | - | - | - | - | - |  | 1 | 0.01 |
| ACCAGC/CTGGTG | - | 1 | - | - | - | - | - | - | - | - |  | 1 | 0.01 |
| ACCATC/ATGGTG | - | - | - | - | - | 1 | - | - | - | - |  | 1 | 0.01 |
| ACCGCC/CGGTGG | 1 | - | - | - | - | - | - | - | - | - |  | 1 | 0.01 |
| AGAGAT/ATCTCT | 1 | - | - | - | - | - | - | - | - | - |  | 1 | 0.01 |
| AGATGC/ATCTGC | - | 1 | - | - | - | - | - | - | - | - |  | 1 | 0.01 |
| AGCAGG/CCTGCT | 1 | - | - | - | - | - | - | - | - | - |  | 1 | 0.01 |
| AGGCGG/CCGCCT | 1 | - | - | - | - | - | - | - | - | - |  | 1 | 0.01 |
