# Supplementary material for: De Novo Assembly of Transcriptome and Development of Novel EST-SSR Markers in Rhododendron rex Lévl. through Illumina Sequencing
Source: Front Plant Sci. 2017 Sep 26;8:1664. doi: 10.3389/fpls.2017.01664 (PMC5622969; doi:10.3389/fpls.2017.01664)

Supplementary Material

De novo assembly of transcriptome and development of novel EST-SSR markers in Rhododendron rex Lévl. through Illumina sequencing

Authors: Yue Zhang, Xue Zhang, Yue-Hua Wang, Shi-Kang Shen*

School of Life Sciences, Yunnan University, Kunming No. 2 Green lake North road Kunming, Yunnan, 650091, China.

***Correspondence author:** Shi-Kang Shen

Supplementary Figure 3 Principal coordinates analysis (PCA) of 20 individuals from 4 populations of *R. rex*

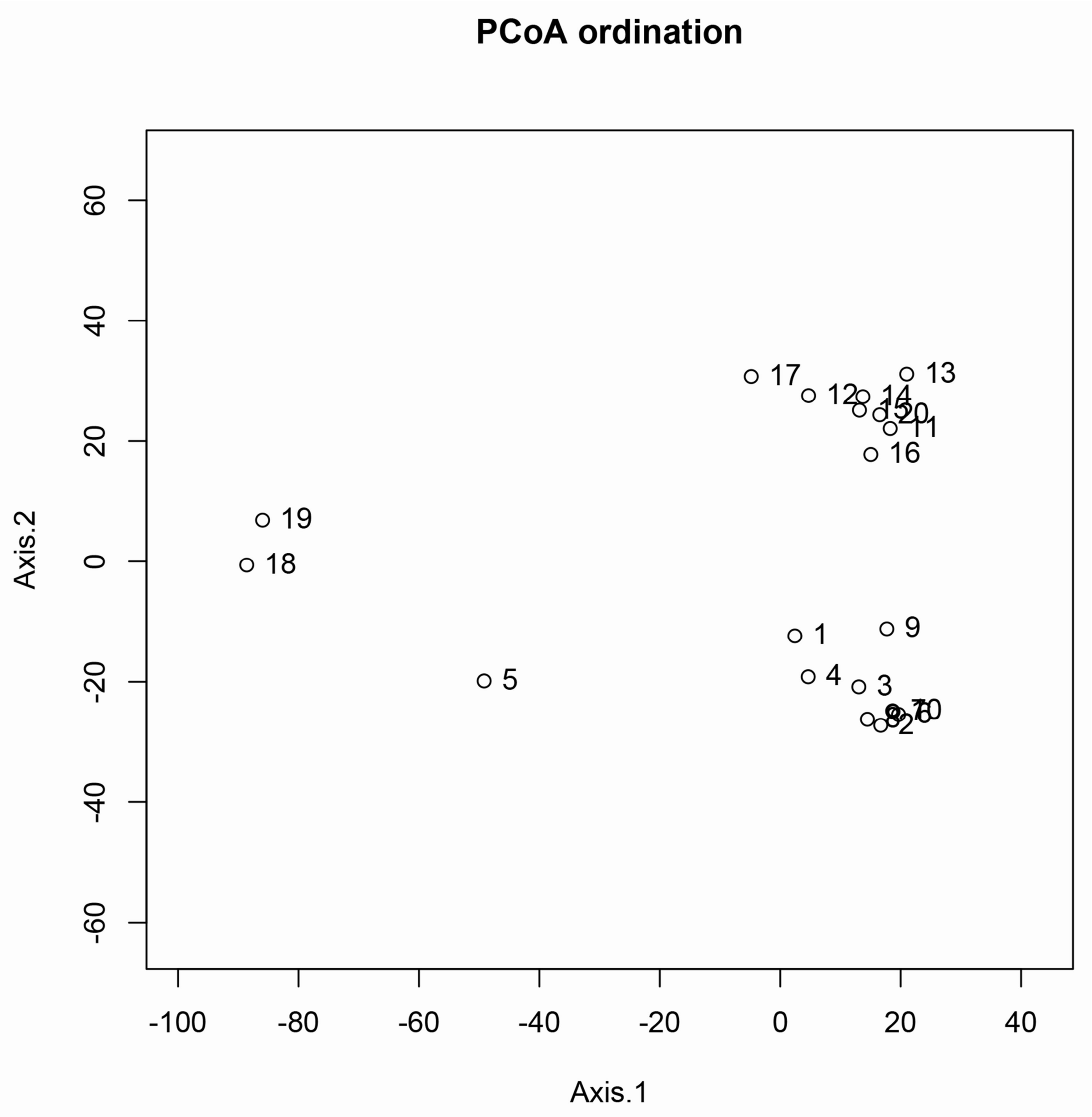

Supplement: Supplementary file 7 [file Image3.pdf]
